# The Influence of Joe Wicks on Physical Activity During the COVID-19 Pandemic: Thematic, Location, and Social Network Analysis of X Data

**DOI:** 10.2196/49921

**Published:** 2024-03-29

**Authors:** Wasim Ahmed, Opeoluwa Aiyenitaju, Simon Chadwick, Mariann Hardey, Alex Fenton

**Affiliations:** 1 Management School University of Stirling Stirling United Kingdom; 2 Business School Manchester Metropolitan University Manchester United Kingdom; 3 School of Knowledge Economy and Management Paris France; 4 Business School University of Durham Durham United Kingdom; 5 Business School University of Chester Chester United Kingdom

**Keywords:** social media, social network analysis, COVID-19, influencers, public health, social network, physical activity, promotion, fitness, exercise, workout, Twitter, content creation, communication

## Abstract

**Background:**

Social media (SM) was essential in promoting physical activity during the COVID-19 pandemic, especially among people confined to their homes. Joe Wicks, a fitness coach, became particularly popular on SM during this time, posting daily workouts that millions of people worldwide followed.

**Objective:**

This study aims to investigate the influence of Joe Wicks on SM and the impact of his content on physical activity levels among the public.

**Methods:**

We used NodeXL Pro (Social Media Research Foundation) to collect data from X (formerly Twitter) over 54 days (March 23, 2020, to May 15, 2020), corresponding to the strictest lockdowns in the United Kingdom. We collected 290,649 posts, which we analyzed using social network analysis, thematic analysis, time-series analysis, and location analysis.

**Results:**

We found that there was significant engagement with content generated by Wicks, including reposts, likes, and comments. The most common types of posts were those that contained images, videos, and text of young people (school-aged children) undertaking physical activity by watching content created by Joe Wicks and posts from schools encouraging pupils to engage with the content. Other shared posts included those that encouraged others to join the fitness classes run by Wicks and those that contained general commentary. We also found that Wicks’ network of influence was extensive and complex. It contained numerous subcommunities and resembled a broadcast network shape. Other influencers added to engagement with Wicks via their networks. Our results show that influencers can create networks of influence that are exhibited in distinctive ways.

**Conclusions:**

Our study found that Joe Wicks was a highly influential figure on SM during the COVID-19 pandemic and that his content positively impacted physical activity levels among the public. Our findings suggest that influencers can play an important role in promoting public health and that government officials should consider working with influencers to communicate health messages and promote healthy behaviors. Our study has broader implications beyond the status of fitness influencers. Recognizing the critical role of individuals such as Joe Wicks in terms of health capital should be a critical area of inquiry for governments, public health authorities, and policy makers and mirrors the growing interest in health capital as part of embodied and digital experiences in everyday life.

## Introduction

### Background

Numerous countries across the world are encountering significant health problems, which are often considered to be a crisis [1]. These problems range from increasing obesity [2] and a high incidence of diabetes [3] to heart problems [4]. The acuteness of such problems is thought to be the consequence of factors including, for example, poor diet [5]. However, they are also identified as being associated with the increasingly sedentary lifestyles that some people lead [6]. A sedentary lifestyle is characterized as one that involves extended engagement in behaviors characterized by minimal movement, low energy expenditure, and significant periods of rest [7].

This study aims to examine the role of a fitness influencer on the social media (SM) platform known as “X.” X serves as a platform for the real-time dissemination of information, public engagement, and discourse. Rebranded in 2023 from “Twitter” (which was the name of the platform when the study was conducted), X allows users to post brief messages, now called “posts,” which can be easily shared through “reposts” and interacted with by a global audience. The fitness influencer under study is Joe Wicks, who is a British fitness coach, TV presenter, and author. Wicks gained considerable public attention during the COVID-19 pandemic through his web-based workout sessions designed to promote physical exercise during lockdowns.

During the first quarter of 2020, most countries globally were affected by the onset of the COVID-19 pandemic [8]. A feature of lockdowns across the world was the compulsory closure of schools. This further heightened worries about physical inactivity during the pandemic, as schools are often the only means some children can engage in physical activity [9]. This often resulted in the physical aspects of school curriculums either being ignored or neglected [10]. Hence, during the early days of the first lockdowns taking place worldwide, there was considerable societal and parental concern about maintaining activity levels. It is within this context that a British fitness coach, Joe Wicks, took the unilateral decision to become the nation’s self-appointed “school PE teacher” (PE refers to physical education). At the time, Wicks had already become a prominent figure on SM, sharing fitness tips and training programs via his YouTube, Instagram, and X accounts.

Given the profound impact that Wicks has had on people’s attitudes and behaviors, we undertook to examine the nature of his SM influence. Specifically, we aim to highlight how his content has moved around a SM network, identify significant other users, determine the shape of Wicks’ network, analyze how people interacted with the content, and identify the geographical spread of content. In so doing, this study’s overall contribution is to how individuals such as Wicks influence others to engage in physical activity.

The policy implications of this study are significant, with health organizations showing interest in this line of work. Although there is some evidence from the literature to indicate that spokespeople and endorsers have played an important role in state-led communications campaigns [11], SM has, therefore, changed the dynamic of relations between governments and populations [12]. We thus assert that this study has significant implications for public health messaging and the way such communications are delivered by governments.

### Overview of Existing Research

In this section, we will review discussions on the formation of social networks, SM, and SM influencers in the context of public health. Furthermore, we highlight research gaps in SM engagement in public health. SM can be described as the collection of digital channels and tools such as Facebook, YouTube, and X with defining characteristics to facilitate engagement [13,14] and encourage user-generated content, social interaction, and real-time collaboration. Specifically, SM use is one of the most popular web-based activities, with an estimated 3.6 billion users worldwide as of 2020, a number that is projected to increase to approximately 4.41 billion by 2025 [15]. Facebook, X, Instagram, WhatsApp, and Google are some of the SM platforms being used every day by millions of people worldwide for accessing and disseminating information [16].

Heinze et al [17] describe SM influencers as individuals who influence behavior to a lesser or greater extent. They can be classed as micro, macro, meso, or mega influencers in terms of their scale of influence, number of followers, and characteristics. SM also enables people to interact from diverse geographical locations. It also provides the opportunity to learn about an audience through conversations. SM not only shapes lives but also influences professional environments [18,19]. The increasing use of SM in health care has enabled its users to access and share information regarding health topics. As such, it has empowered users to take control of their health care needs [20].

Public health organizations need to further harness SM channels to identify what people care about and are discussing to ensure an informed communication strategy [21]. Despite the fact that the various conversations on the possible relevance SM could have in influencing health behaviors and outcomes [18,22], oftentimes, public health organizations use SM as a 1-way broadcast and mass dissemination of information rather than engaging audiences in interactive information-sharing dialogue [14,23,24]. The increased use of SM is prevalent, “People have discovered ways to use SM to impact their health, their community’s health, and public health, much of which has gone entirely unstudied” [25]. Bruno Latour has produced significant work on the nature of truth, particularly in science and public health. Latour argues that truth is not a fixed or objective thing but rather is constructed through social interactions. This means that what we consider true is a matter of facts and the relationships between people, objects, and ideas. Latour’s work has important implications for the study of influencers—particularly the impact of digital health contexts [26]. If truth is not fixed, then the influence of an influencer may not be solely based on their expertise or trustworthiness. Rather, it may also be influenced by factors such as the influencer’s social network, their ability to connect with their audience, and how they frame their messages.

SM is useful for identifying the key public health influencers and driving web-based conversations on health and well-being topics that are valuable and meaningful to them. Influencers can be classed as both organizations and individuals [27,28] with qualities such as credibility, expertise, and enthusiasm [29]. Influencers are also persistent in convincing consumers or driving conversations on a topic. Among other interesting descriptions of SM influencers, they are viewed as those who have built a sizeable social network of followers or as a third-party endorser that shapes audience attitudes using SM such as blogs, posts, and so on [30-32]. In their early assessment of SM’s impact on health research, Moorhead et al [33] discovered that SM can improve health care by providing a platform for public engagement, patient interaction, and health-professional communication regarding health-related issues. These interactions have the potential to promote health. In addition, the authors speculate how SM can enhance health communication. However, the authors caution that it is essential to carefully monitor the quality and veracity of information shared on SM platforms while protecting the privacy of users. These results align with those of studies conducted on health determinants.

The literature discusses various challenges in SM engagement for public health. The need for studies with larger sample sizes and more robust methodologies to study SM algorithms and behavior is crucial [33-35]. A literature gap also exists relating to the effectiveness of SM engagement on public health outcomes and how these impacts change individual behavior, altering public attitudes and influencing public health policy [14,25,36]. Increasingly, and certainly, in response to national lockdowns for COVID-19, SM serves as a conduit for health-related information and support. Such digital use by health consumers, some of whom are motivated to join online community groups to help cope with long-term health conditions, has been linked to increases in social support and coping and support for mental health [37,38]. For example, Hardey’s [39] and Hjorth and Lupton’s [40] research has demonstrated how the combination of health information on SM and the use of self-tracking technologies for managing chronic conditions is associated with higher levels of health sharing between intimate partners and new forms of social support, as well as improved engagement with health practitioners. In addition, Weinstein et al [41] found for some young men that the use of SM support groups led to reductions in feelings of loneliness and suicidal thoughts. Finally, Dowrick et al’s [42] study of digital connections between UK health care workers during the COVID-19 pandemic showed the potential use of SM to support care interactions and increase patient social capital. Patient social capital refers to the resources that a patient has access to through their social network, such as emotional support, financial assistance, practical help, and information [43].

While this body of work paints an inspiring image of the impact of digital networks on health consumers, 2 significant areas in the literature require further investigation. The first involves prior studies focusing on the influence of digital networks on individual health consumers or specific health concerns and evaluating long-term perceptions of change in response to certain types of information (including well-known individuals). Monitoring this type of health behavior change covers broader networks, such as caregivers, who require assistance in caring for and coping with patients [44]. The second area that deserves investigation is the interaction between digital media consumption, social capital [45,46], and intended health outcomes [47]. Despite the fact that there has been little research on the effectiveness of health influencers’ reach and the means by which they might promote health change, a number of studies have looked at how digital content use affects social capital—and, as a result, health outcomes. Natural language processing was used in 1 study to analyze X text in order to assess how fitness affects the development of social capital [48]. Another study compared multiple SM platforms (YouTube and Instagram) with health care professionals, fitness influencers, and healthy food influencers in order to quantify engagement [49].

Previous studies have hypothesized that how consumers interact with or have access to digital health content improves their sense of social support, which influences well-being, which affects social capital and health outcomes [50]. This study aims to establish the extent to which Joe Wicks had an impact on health during the pandemic. By analyzing network reach and themes, we hope to analyze representations of health and pictures of fitness as interpreted through digital engagement. By doing so, we can better examine digital manifestations of “health” in everyday life—the components of and settings for embodied fitness activities. Furthermore, by determining Wicks’ influence, we aim to assess whether this information was effective in persuading people to engage in physical exercise during the pandemic. This study adds to the growing body of research on governments’ neoliberal health objectives and the depiction of fitness personalities in the perception and engagement with health and fitness. The study adds empirical understandings to the study of fitness and health influencers while also engaging critically with literature. To make SM engagement more effective in a crisis, influencers or public health communicators should be able to respond and engage with the audience in real time [51].

## Methods

### Data Retrieval

The X Academic Track application programming interface was used to retrieve posts from March 23, 2020 (T00:00:00Z) to May 15, 2020 (T23:59:00Z). This time period covered a time that included some of the strictest lockdown policies during the pandemic in the United Kingdom. In total, 290,649 posts were retrieved. The keywords “Joe Wicks” or “thebodycoach” were used to retrieve posts, and this included users who were replied to or mentioned in posts. The term “thebodycoach” was used as it is the X (Twitter) user handle of Joe Wicks. X is the most open network and, therefore, lends itself well to social network analysis (SNA) and SM research [52]. The method has been widely used in recent research to study COVID-19 disinformation [53-55].

### Data Analysis (SNA and Thematic Analysis)

#### SNA

**Figure 1:**
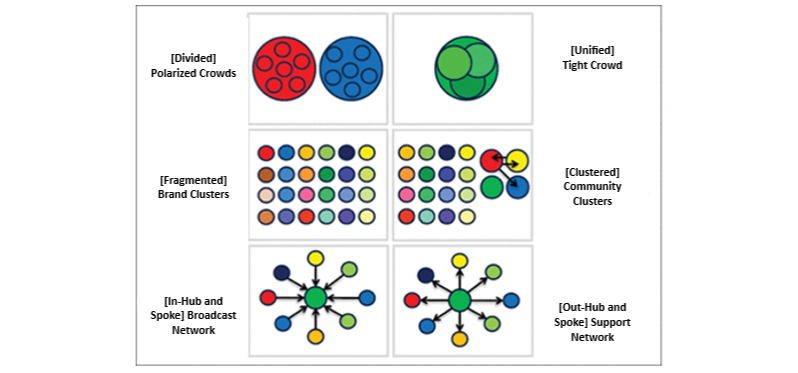
Six types of X networks (simplified form). Sources: Smith et al [57] and the Social Media Research Foundation.

A systematic random sample of data (5%) was entered into NodeXL Pro for further SNA analysis. Numerous research studies have successfully used NodeXL Pro for analyzing SM data using SNA to develop new knowledge [56]. Moreover, previous empirical research has identified 6 types of X network structures that topics on X follow, as shown in Figure 1 [57]. This study used an SNA approach to answer the research questions.

The information flow within the SM space involves a few types of engagements of users with content such as following, subscribing, sharing, and reposting [58]. SM users form a pattern of connections viewed as social networks. For instance, on X, connections are formed when users mention, like, reply, or repost other users, which reveal the shape of their network [59]. The structure of a network captures patterns of information flow, such as measurements of centrality, degree, and betweenness [60,61]. Centralization captures the degree of aggregated connections around a few actors in a network. Centrality depicts the hierarchy of information flow and allows the identification of influential users in a network. Research on X has found that these information flows revolve around 6 kinds of X SM network structures: polarized crowds (divided), tight crowds (unified), brand clusters (fragmented), community clusters (clustered), broadcast networks (in-hub and spoke), and support networks (out-hub and spoke) [57], which were provided above.

#### Thematic Analysis

Data were then analyzed using thematic analysis [62] until thematic saturation occurred [63]. A sample of data (n=400) was then used to project the frequency of themes across the data which would reflect the sample overall. Braun and Clarke’s [62] thematic analysis consists of 6 main steps aimed at identifying and analyzing patterns or themes. The process began with becoming familiar with the data through repeated reading and initial note-taking. The second step was to generate initial codes from the data. Coding is the process of tagging or highlighting segments of the data that appear interesting or relevant to the research question. In the third step, the focus was on searching for themes by collating all the coded data. The fourth step was to review these potential themes to ensure they have enough data to support them and that they are distinct from one another. The fifth step was to define and name themes. This involved developing a detailed analysis of each theme, determining what aspect of the data each theme captured and what was interesting about them. Finally, the sixth step was to write up the results.

### Betweenness Centrality

The betweenness centrality metric can be used to detect influence within a network. We can express the betweenness centrality of a node “v” as follows:



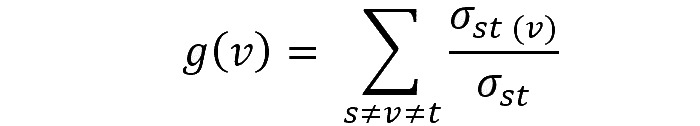



**Figure 2:**
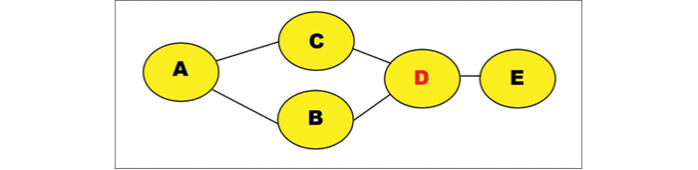
Betweenness centrality example.

In relation to the above, σ_st_ is the total number of shortest paths from node s to node t, and σ_st_(v) is the occurrence of those paths that pass through v (but not where v is the end point). The metric is commonly applied to SM platforms to detect influential users [64]. This is because betweenness centrality can identify the important users within a network based on how much they act as a bridge between different parts of a network. We can think of this in another way. That is, if we were to remove select users with a high betweenness centrality, this would disrupt the entire network. This is further represented in Figure 2 below, which shows “D” in red as the most influential node because it has a unique link to node “E.”

### Ethical Considerations

The study gained ethical approval from Newcastle University (Ref: 26055/2022). The study was observational using SM data. However, care was taken to not draw attention to individual users, and SM posts and user handles were anonymized within the manuscript.

## Results

### Results of SNA

**Figure 3:**
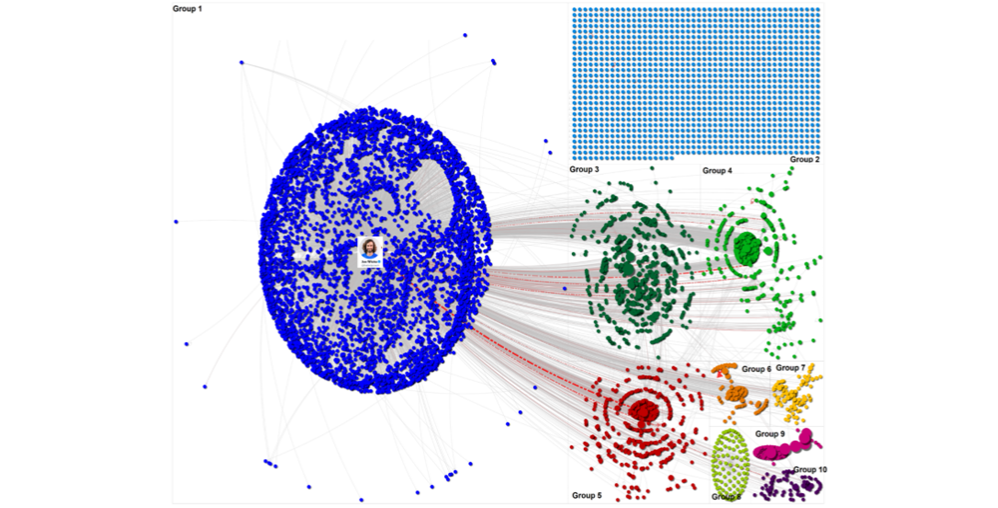
Examining the top 10 largest groups on X posting about Joe Wicks (March 23, 2020, to May 15, 2020).

Figure 3 provides an overview of the network’s top 10 groups, and each group is labeled from the largest (group 1) to the smallest (group 10). The network shows Wicks’ influence across the top 10 groups of users. We can see that Wicks’ reach stretched across several groups due to the number of lines pointing out the group to which Joe Wicks belongs, as this indicates users in other groups were reposting and mentioning Wicks’ content. Groups 2 to 10 highlight how several smaller subcommunities have formed around Wicks’ content, led by other influencers that are listed in Table 1.

**Table 1:** Overview of the key influential users.

Rank	User	Betweenness centrality	Followers	Location
1	Joe Wicks	245,642,386	421,565	England
2	James Corden	2,746,155	10,959,093	California
3	Citizen	2,715,772	797	England
4	Citizen	2,559,876	2158	Derbyshire
5	Citizen	2,528,820	4875	United Kingdom and South Africa
6	Freelance writer, editor, and author	2,504,078	10,620	N/A^a^
7	Citizen	1,749,337	9	N/A
8	Citizen	1,402,979	182	England
9	Math teacher	1,311,087	4053	England
10	Citizen	1,284,940	532	N/A

^a^N/A: not applicable.

### Results of Betweenness Centrality

Table 1 provides an overview of the most influential users (ranked by betweenness centrality) and provides insights into the rank, user type, betweenness centrality score, number of followers, and location.

Joe Wicks was among the most impactful, but this is not surprising and potentially an artifact of the data collection (as the user-handle of Joe Wicks and search string was used to obtain data). James Corden is in second place, with 3.6 million followers. James Corden is an English actor, comedian, singer, writer, producer, and television host. He is known in the United Kingdom for cowriting and appearing in the BBC sitcom Gavin and Stacey. In the United States, Corden gained prominence for hosting The Late Show with James Corden, a late-night television program broadcast on CBS from 2015 to 2023.

The list of users also includes several individuals labeled as “Citizens” who were ordinary public members located in areas like England and Derbyshire. One such user has ties to both the United Kingdom and South Africa. These users had varying numbers of followers, ranging from 9 to 4875. Other users included a freelance writer, editor, and author with 10,620 followers, as well as a math teacher from England with 4053 followers. Both have managed to accumulate a noticeable following despite their different professions. The users who are not celebrities, such as the freelance writer as well as the math teacher from England, have still managed to garner a sizable following. This suggests that they have been posting engaging content that has captured the attention and imagination of their audience.

### Time Series Analysis

**Figure 4:**
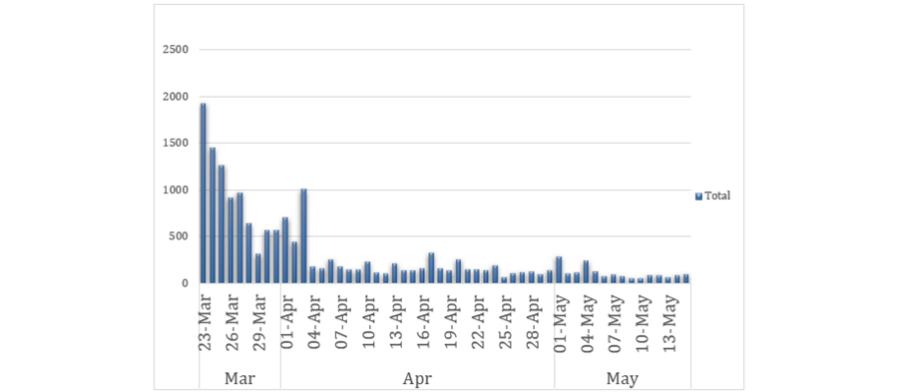
Time series analysis of X activity related to Joe Wicks (March 23, 2020, to May 15, 2020).

Figure 4 provides a time series analysis of posting across the 54-day period. Most of the traffic appears to peak during the first phases of the lockdown, where Wicks was actively promoting his live streams.

### Results of Thematic Analysis

#### Overview of Themes

To set the context for the thematic analysis, a key post was sent by Joe Wicks on March 19, 2020, that kick-started the discussions, the post noted:

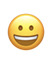
 PE WITH JOE starting Monday morning at 9am on my YOUTUBE channel: The Body Coach TV 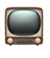 Please please share this with as many people as you can 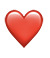 Our kids need this more than ever. Share it on your stories, your wall, your Twitter, whatsapp and school newsletters 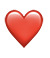

This post called for and encouraged a YouTube fitness session run by Joe Wicks to be shared by others. Wicks highlighted that children would benefit the most from the videos and encouraged users to share the information widely, including school newsletters. The post above was quoted and reposted across March, and based on the number of reposts and likes, it appears this was a very successful approach in spreading the word of the online class.

Table 2 provides an overview of the key themes, their description, and examples of posts for each thematic heading. The most frequent type of posts was sent by parents who were showcasing how their children were engaging with the exercise activities from Wick live streams.

**Table 2:** Overview of key themes and description.

Theme	Description	Examples	Frequency, n (%)
Children taking part	Parents shared posts with images, text, or video showing children taking part and showing appreciation to Joe Wicks.	My children are absolutely LOVING their workouts! [VIDEO]	150 (37.5)
Other commentary and news articles	News articles on Joe Wicks and other general nonspecific commentary.	I wonder if there could be a workout for elderly and disabled who can’t move as much?	110 (27.5)
Encouraging others to join in	Users encouraged others to take place by posting, reposting, and activity mentioning other users.	Hey, everyone! Be sure to get involved with this! [LINK]	90 (22.5)
Schools	Schools would post to encourage pupils to take part in the exercises.	PE classes just like school! Be sure to join in! [LINK]	50 (12.5)

#### Theme 1: Children Taking Part

In this theme, parents would share posts with images, text, or videos showing children taking part in Joe Wicks–related activities to express appreciation for Joe Wicks.

For instance, one user noted:

My children are absolutely LOVING their workouts!

Link to VIDEO of children exercising

Another user would go on to note:

I'm super proud of my children for taking part in Joe Wicks for their daily exercises

Image of Children

The theme showed that parents and caregivers observed the positive effects of physical activity and routine in promoting a sense of mindfulness among families and households that were struggling to manage their time effectively during the lockdown. This is consistent with the instant interconnectedness that SM facilitates and the influence of prominent public figures like Joe Wicks in promoting a sense of collective unity during the pandemic. This finding is consistent with the observations made by Zhu et al [65] and Durau et al [66] about the use of SM as a platform for disseminating narrative-based health content and amplifying health communication.

#### Theme 2: Other Commentary and News Articles

This theme largely contained news articles on Joe Wicks and other general nonspecific commentary. For instance, 1 comment from a user was as follows:

I wonder if there could be a workout for the elderly and disabled who can't move as much?

There was a wide range of news articles commenting on the overall popularity of Joe Wicks, and additionally, media began to report on future ambitions; for instance, 1 post linked to a Daily Mail article that noted:

Joe Wicks Targets the US! The Highly Driven Body Coach Celebrity Plans to Conquer America

The theme reinforced the public interest in health by exploring how the media reported this. More broadly, this theme engages with the proliferation of media coverage and speculation about the impact of this on health. The literature has largely focused on misleading media coverage of COVID-19, particularly discrimination and its impact on human rights [67]. However, alongside these important global social issues, there is also a sense of frivolity in the reporting and reaction to such articles on SM, which seeks to underscore the notoriety of public figures like Wicks in order to establish a global health narrative around exercise.

#### Theme 3: Encouraging Others to Join in

In this theme, users encouraged others to take place by posting, reposting, and actively mentioning other users. For instance, a user posted:

Hey, everyone! Be sure to get involved with this!

LINK to Joe Wicks Content

Other users were more lively and shared their own images; for instance, one user posted:

I've prepared my disco diva attire for the Joe Wicks gathering tomorrow. Have you chosen your ensemble?

Image of user in disco attire

The theme demonstrated the interconnectedness within households in response to Wicks’ exercise activities. The literature suggests that establishing connections can positively impact home dynamics, facilitate individuals’ willingness to discuss personal health concerns, and initiate sustained improvements in individuals’ health behaviors [68,69].

#### Theme 4: Schools

In this theme, schools would post to encourage pupils to participate in the exercises. For instance, 1 school account would post:

PE classes just like school! Be sure to join in!

LINK

Another school posted:

Anyone for Physical Exercise featuring Joe Wicks?

The above post received several replies from parents who would share images of their children taking part and interacting with the school.

The theme highlighted the importance of health interactions and education, as schools played a vital role in advocating for children during the COVID-19 pandemic. Other researchers have also provided evidence of the transformative benefits and impact of education on health. Additionally, there is ongoing speculation about the global consequences of school closures and the effectiveness of information-seeking behaviors and public health messages following the reopening of schools [10,70,71].

Beyond the risks of COVID-19, the themes above also underscore the interest in more light-hearted and enjoyable aspects of health, particularly activities that households can do together. Common across the themes was the desire for connection and the impact of global interactions in supporting public health initiatives.

### Results of Location Analysis

**Figure 5:**
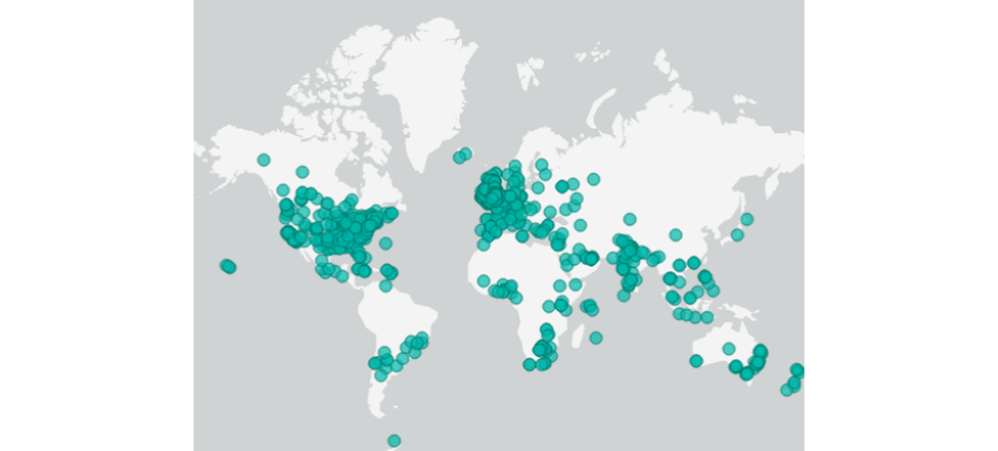
Geographical overview of users on X related to Joe Wicks (March 23, 2020, to May 15, 2020).

**Figure 6:**
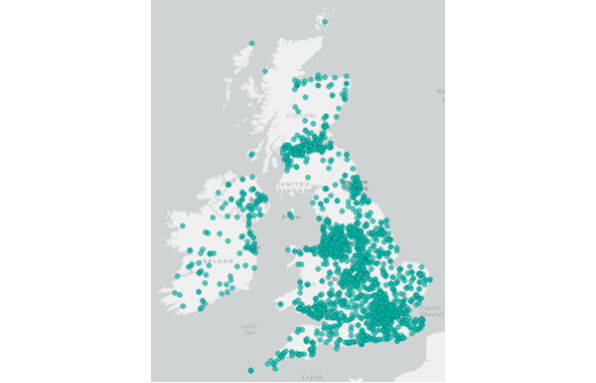
Posts across the United Kingdom related to Joe Wicks (March 23, 2020, to May 15, 2020).

**Figure 7:**
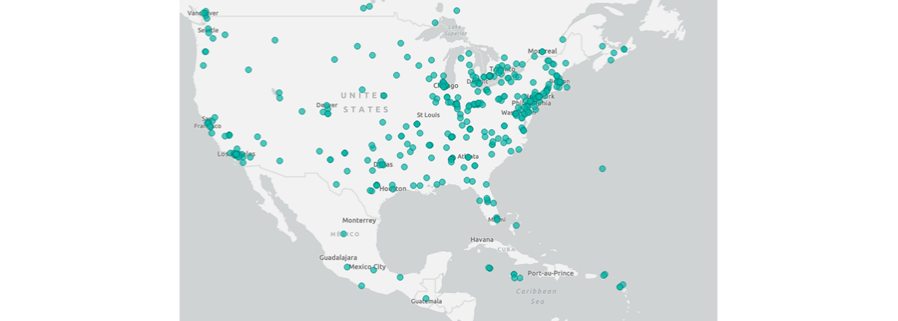
Posts across the United States related to Joe Wicks (March 23, 2020, to May 15, 2020).

Figures 5-7 show a range of maps that document X activity around the world. The cyan-colored dots represent an X interaction (such as a post, repost, or quote post). Figure 5 provides a visual overview of the range of locations that were posted during this time. Due to the borderless reach of X, users around the world were engaging with content shared by Wicks.

Figure 6 provides an overview of X activity in the United Kingdom. It shows activity across the United Kingdom, with users clustered in Scotland and England.

Figure 7 provides insight into activity from X in the United States. It shows a geographical spread of X users across various states.

## Discussion

### Principal Findings and Implications

This study examined how much Joe Wicks, a fitness influencer, impacted health and health routines during the COVID-19 pandemic. We found that Wicks was a key influencer in motivating people to stay active and fit during this time. His network of influence was extensive and complex and included numerous subcommunities. We found that fitness influencers can create networks of influence exhibited in distinctive ways.

Wicks’ SM accounts were being viewed by millions of people—domestically, internationally, and globally [72]. Indeed, reports of his workout sessions commonly began referring to him as “the nation’s PE teacher.” What is clear is that throughout the pandemic lockdown, Wicks had a profound effect on attitudes and behavior toward physical fitness, influencing large numbers of people to engage with and in physical activity to the extent that Britain has never seen before (at least in recent times). Wicks was awarded an MBE (Member of the Order of the British Empire) in 2020 for his live streams during the pandemic and a Guinness World Record for the most views on a fitness workout live on YouTube, with over 950,000 viewers [73].

Our findings support the hypothesis that Wicks positively impacted public health during the pandemic. His content encouraged people to engage in physical activity, an essential component of a healthy lifestyle. Such nudges toward health activities are especially important during times of crisis, when people may be more likely to neglect their health.

We found that ordinary citizens sharing content created by Joe Wicks were the key influencers and bridges in his network. Our results show that Wicks’ network of influence resembled a broadcast network with several smaller subcommunities. A qualitative analysis of posts further highlighted how Wicks’ content had encouraged younger people to continue with physical activity, which likely improved their overall health and well-being.

Our analysis shows that Joe Wicks was likely to have influenced physical exercise and activity across the world during the pandemic. Hence, the underpinning central tenant of this study is confirmed. As such, we conclude that SM, in this case, X, can serve both as an influencing platform in its own right and as a channel through which individuals can influence [74]. The relevance of social capital in relation to health is now well recognized in the social sciences, and theoretically informed studies on fitness influencers’ promotion and portrayal of “healthy” bodies is a developing area of interest for health sociology and ill health studies [75,76]. With its long-standing empirical interest in social capital, health sociology is beginning to pay attention to the personalities and figures who have emerged because of the shift to neoliberal health policy. However, because this work is relatively new [77], important themes and areas of investigation are still being developed.

Our study has broader implications beyond the status of fitness influencers. Recognizing the critical role of individuals such as Joe Wicks in terms of health capital should be a critical area of inquiry for governments, public health authorities, and policy makers and mirrors the growing interest in health capital as part of embodied and digital experiences in everyday life. This is an especially important observation to make in Western cultural settings; given cynicism about government and the role of politicians, trust between them and the population is in question. Indeed, we suggest that such is this cynicism that some populations choose not to believe or engage with government messaging. On this basis, we contend that this study is an especially important one because it highlights the opportunities for governments, health officials, and policy makers to effect behavioral change. That is, rather than state entities seeking to communicate with and engage populations, we believe that SM influencers could be a more effective way of inducing behavioral change (on behalf of states and countries).

In addition to influencers and their influence, this study also highlights the role of significant others in SM networks. If Wicks has been the SM engine driving influence, then a community of other SM users has been the fuel that has built and sustained Wicks’ influence. Given the scale of the SM ecosystem, we speculate that only a small number of what we label “ultra-influencers” are likely to have the reach and power to affect attitudinal and behavioral change (the likes of Hollywood actors and international music stars come to mind). As such, while we acknowledge the reach and power of Wicks, we nevertheless believe that his overall influence is an outcome of the aggregated influence of several others. Moreover, there is scope here to combine the ultrareputation of influencers such as Wicks with health technologies to further help individuals and households to make and sustain changes [78].

### Limitations and Future Research

Our mixed methods approach offers significant analytic strength, providing a more robust understanding of Wicks’ presentation and influence. SNA reveals how Wicks’ presentation took hold within health narratives, while thematic analysis provides nuance to its nature.

Wicks’ fitness influencer status has 2 leading roles: educator and motivator for health activities. The pandemic’s national and global policy changes prevented a more robust longitudinal design with the thematic data. Future research could investigate how other fitness influencers adapt their presentation across multiple platforms and situations over time. Additionally, Wicks’ daily fitness routines encapsulate his health strategy during a crisis rather than more robust health resources outside the pandemic. Therefore, while the analysis of Wicks’ daily fitness routines allows us to deduce different types of engagement, we cannot be sure of the levels of interest in the depiction of fitness and how health is presented in its own right when it is part of influencer messaging.

We focused our research on Wicks’ influencer-style performance of fitness in his shared content. Our findings suggest that the content allowed Wicks to successfully engage in a unique form of impression management applied to fitness and health strategies. However, Wicks’ followers can also perform in healthy ways; this study provides a limited overview of how this manifested among Wicks’ followers. We suspect that followers of fitness influencers would likely be qualitatively different. Because fitness tropes and health societal structures are embedded in individuals’ lived experiences, how fitness is experienced would differ toward different aims and be expressed differently. We suggest this is a fruitful area for future research. Additionally, our mixed methods approach does not allow us to directly question followers about the broader impacts of their engagement with Wicks’ content. Future interview-based research might seek to deepen the meanings and themes here. Future research might also investigate the role of fitness influencers in shaping the health performance of followers on SM.

A further limitation of the study is the timeframe examined. The research captures conversations on X related to a specific snapshot in time. Therefore limiting the ability to reflect changes in public behavior or opinion over a longer period. Consequently, the findings may not be generalizable over different periods. However, they would provide a useful comparison point for future studies examining other periods. Another limitation involves the challenges of conducting a qualitative analysis of brief posts. X’s character limit at the time (280 characters) means that deep and nuanced insights may not emerge compared with interview-based data. Furthermore, the brevity of posts means that users often draw upon abbreviations, hashtags, and other condensed forms of communication that are not easily amenable to detailed qualitative interpretation. Furthermore, the study focused solely on X data, which could introduce biases because it may not accurately represent a larger population. X users may have specific demographic characteristics that are not fully representative of the population being studied. A further limitation of the study is the focus on X interactions as the primary metric for gauging the effectiveness of motivating physical exercise during the pandemic. While this approach offers insights into public engagement, it may not fully capture the nuanced behaviors and long-term commitments of individuals who were influenced but did not express this online. For instance, some individuals may have been motivated to exercise regularly without posting their experiences on SM, while others may have expressed intent online but did not follow through offline. Future research could benefit from a multidimensional approach that combines SM analytics with in-person or survey-based methods to provide a more comprehensive understanding of the impact.

### Conclusion

The fact that 1 person can influence the behavior of another is nothing new. What has changed in the last 2 decades is the rise of SM platforms like X. As a result, the nature of influence and the role that individuals and groups play as influencers has shifted. As a result, there is developing literature on this topic. We have contributed to this body of work by analyzing Joe Wicks’ influence. This study demonstrates that individuals like Wicks can influence behavioral change. This alone justifies additional research in both related and unrelated circumstances concerning the impact of key persons of influence on health behavior change and fitness. Platforms such as X and popular accounts like “@thebodycoach,” find considerable reach and influence through strong reputational links (for instance, increased user engagement or even trending topics).

There are various methods for gaining influence through SM platforms; in this study, Wicks stood out as a central node in a broadcast network. In turn, his impact has been maintained by the second wave of influencers. This influence hierarchy creates exciting research opportunities in this and other contexts. Additionally, meta-comments on health and fitness in this example reflect a shift in user motivation, driven in part by the feeling of digital engagement but also by the ability to impact real-time health behavior change during the Joe Wicks live-streamed training sessions. The collectivized activity of this type is an intriguing and significant area for research, combining digital and offline activities aimed at behavior modification and, we argue, social capital, but it requires additional investigation, and we conclude with such a call to action for other researchers working in this field.

Finally, we have seen how Wicks has been able to influence behavior in ways that governments and health officials frequently cannot. As a result, we urge that researchers and practitioners focus more on influence and influencers in order to improve the effectiveness of public health policies and communications. After all, if an online fitness instructor can persuade millions to exercise on a daily basis, other such influencers should be able to have similar effects in a variety of other contexts.

## Abbreviations

NLP: natural language processing
SM: social media
SNA: social network analysis

## Data Availability

The data sets generated or analyzed during this study are available from the corresponding author on reasonable request.
